# Salt Stress Modulates the Landscape of Transcriptome and Alternative Splicing in Date Palm (*Phoenix dactylifera* L.)

**DOI:** 10.3389/fpls.2021.807739

**Published:** 2022-01-20

**Authors:** Zhongliang Xu, Ning Zhang, Haiquan Fu, Fuyou Wang, Mingfu Wen, Hailong Chang, Jiantao Wu, Walid Badawy Abdelaala, Qingwen Luo, Yang Li, Cong Li, Qinnan Wang, Zhen-Yu Wang

**Affiliations:** ^1^Sanya Research Institute, Chinese Academy of Tropical Agricultural Sciences, Hainan, China; ^2^Coconut Research Institute, Chinese Academy of Tropical Agricultural Sciences, Hainan, China; ^3^Institute of Nanfan & Seed Industry, Guangdong Academy of Sciences, Zhanjiang, China; ^4^Zhanjiang Sugarcane Research Center, Guangzhou Sugarcane Industry Research Institute, Guangzhou, China; ^5^Central Laboratory for Date Palm Research and Development of Agriculture Research Center, Giza, Egypt

**Keywords:** date palm, alternative splicing, salt stress, serine-arginine-rich proteins, signal transduction

## Abstract

Date palm regards as a valuable genomic resource for exploring the tolerance genes due to its ability to survive under the sever condition. Although a large number of differentiated genes were identified in date palm responding to salt stress, the genome-wide study of alternative splicing (AS) landscape under salt stress conditions remains unknown. In the current study, we identified the stress-related genes through transcriptomic analysis to characterize their function under salt. A total of 17,169 genes were differentially expressed under salt stress conditions. Gene expression analysis confirmed that the salt overly sensitive (SOS) pathway genes, such as *PdSOS2;1*, *PdSOS2;2*, *PdSOS4*, *PdSOS5*, and *PdCIPK11* were involved in the regulation of salt response in date palm, which is consistent with the physiological analysis that high salinity affected the Na^+^/K^+^ homeostasis and amino acid profile of date palm resulted in the inhibition of plant growth. Interestingly, the pathway of “spliceosome” was enriched in the category of upregulation, indicating their potential role of AS in date palm response to salt stress. Expectedly, many differentially alternative splicing (DAS) events were found under salt stress conditions, and some splicing factors, such as *PdRS40*, *PdRSZ21*, *PdSR45a*, and *PdU2Af* genes were abnormally spliced under salt, suggesting that AS-related proteins might participated in regulating the salt stress pathway. Moreover, the number of differentially DAS-specific genes was gradually decreased, while the number of differentially expressed gene (DEG)-specific genes was increased with prolonged salt stress treatment, suggesting that AS and gene expression could be distinctively regulated in response to salt stress. Therefore, our study highlighted the pivotal role of AS in the regulation of salt stress and provided novel insights for enhancing the resistance to salt in date palm.

## Introduction

Date palm (*Phoenix dactylifera* L.) is an important fruit crop in several arid and semiarid countries in North Africa, the Middle East, and Central America. Dates play a pivotal role in the economy, society, and environment of those countries, and regard as the main income source and staple food for local populations (Martis et al., 2020). The date palm trees have the ability to grow under the severer climatic conditions, such as very hot and dry climates, and even in close proximity to the seashore that contained large amounts of salty and alkaline soils (Yaish and Kumar, 2015). Therefore, the date palm trees were supposed to be drought- and salt-tolerant plants and have evolved a series of mechanism for their adaptation.

A high level of salt results in severe soil problems, and high salinity in soil impairs the growth due to the toxicity of Na^+^ and other ions (Shrivastava and Kumar, 2015). Plants have evolved several adaptations to deal with high salinity by regulating the ion influx and efflux at the plasma membrane, ion compartmentation in vacuoles, and maintaining the osmotic balance (van Zelm et al., 2020). Salinity causes plants to accumulate the compatible solutes, such as sugars and sugar alcohols and the synthesis of amino acids and amines (Ma et al., 2020). Meantime, high salinity results in the overstimulated reactive oxygen species (ROS), and plants have developed the enzymatic and non-enzymatic strategies to eliminate the excessive ROS to maintain redox homeostasis (AbdElgawad et al., 2016; Hasanuzzaman et al., 2021). In addition to trigger this cellular adaptation, expressions of many stress-responsive genes, such as ionic transporters, transcription factors, and protein kinases have been regulated by salt stress (Zhu, 2016; Yang and Guo, 2018b; Zhang et al., 2021).

Regulation of gene expression was involved in a multi-step process that is highly interrelated and modulated at diverse levels. Transcription factors bind to the specific DNA sequences and recruit RNA polymerases for RNA synthesis (Nikolov and Burley, 1997; Schier and Taatjes, 2020; Gajos et al., 2021). Once RNA precursors are formed, nascent mRNA precursors associate with ribonucleoprotein complexes and mediate diverse RNA processing reactions, such as alternative splicing (AS), capping and polyadenylation, mRNA transport, mRNA stability, and translation of the functional mRNA (Lee and Rio, 2015; Shang et al., 2017; Bernardes and Menossi, 2020). Later, mRNAs undergo RNA decay by the RNA exosome (Sorenson et al., 2018). AS is mediated by the spliceosome including five small nuclear ribonucleoprotein particles (snRNPs) and hundreds of non-snRNP proteins (Lee and Rio, 2015; Shang et al., 2017; Martin et al., 2021). Numerous studies reveal that salt stress promotes the aberrated splicing of stress-responsive genes and spliceosome components which affects the plant salt tolerance (Ding et al., 2014; Feng et al., 2015; Gu et al., 2018). Manipulation of spliced or unspliced gene isoforms can maintain the plant tolerance to salt and other stresses (Cui et al., 2014; Feng et al., 2015; Wang et al., 2015, 2020; Gu et al., 2018; Albaqami et al., 2019; Lee et al., 2020; Li et al., 2021; Weng et al., 2021). Therefore, manipulation of gene isoforms has a critical role for increasing the plant adaption to salt and other stresses.

With the release of date palm genome (Al-Mssallem et al., 2013), transcriptomic analysis of date palm tree in response to salt stress was conducted, and a large number of differentiated genes were observed in the tissues (Radwan et al., 2015; Yaish et al., 2017). However, genome-wide study of AS landscape remains unknown under salt stress conditions. In the present study, the global dynamics of AS were investigated in the date palm trees that were exposed to high salinity by pair-end RNA sequencing. We first identified amounts of differentiated genes under salt stress in comparison with previous studies (Radwan et al., 2015; Yaish et al., 2017). Gene expression analysis confirmed that the salt overly sensitive (SOS) pathway genes were involved in the regulation of salt response in date palm, which is consistent with the physiological analysis that high salinity affected the Na^+^/K^+^ homeostasis and amino acid profiles of date palm that resulted in the inhibition of plant growth. Importantly, many differentially alternative splicing (DAS) events were found under salt stress, and some splicing factors were abnormally spliced under salt, suggesting that AS-related proteins might be involved in regulating the salt stress pathway. Therefore, our study highlights the pivotal role of AS in the regulation of salt stress and provided novel insights for enhancing the resistance to salt in date palm.

## Materials and Methods

### Plant Materials and Growth Conditions

Seeds of the date palm (*P. dactylifera* cv. Mabroom) were planted in the pot containing the peat moss. The trees were maintained in the greenhouse under natural sunlight. The temperature and humidity were maintained at 28 ± 3 °C and 40% during the daytime and 23 ± 2 °C and 60% at night. After 6 months, a similar size of date palm tree was chosen, and then transferred to the container that contained a different concentration of NaCl solution. Plants were photographed at the indicated time after salt stress treatment. To collect the salt-treated samples for RNA-seq, the leaves with the same position were harvested under 1,000 mM NaCl for indicated time as apparent salt stress phenotype, such as yellowish and curled leaves was observed in date palm, and frozen in liquid nitrogen for subsequent analysis.

### Measurement of Na^+^, K^+^, and Amino Acid Content

For Na^+^ and K^+^ content measurements, the leaves samples were collected at the indicated time, and then, dried for 48 h at 80°C. Na^+^ and K^+^ from the leaves were extracted and measured using inductively coupled plasma (ICP)-atomic emission spectrometry based on previous protocol (Jarvis and Jarvis, 1992).

Amino acid profiling was performed as described before with brief modification (Williams et al., 2007). The harvested leaves were lyophilized for 1 week. About 100 mg of lyophilized powder was used for metabolite profiling by gas chromatography–mass spectrometry (GC-MS). Peaks were manually annotated, and the ion intensity was determined by the standard amino acids as a reference.

### RNA Extraction, Library Construction, and Sequencing

Total RNAs were extracted from seedlings with or without salt stress treatment by using the RNAprep Pure Plant Kit (Tiangen Biotech, China). Polyadenylated RNAs were isolated, chemically fragmented, and synthesized, and the cDNA fragments were then amplified by PCR using adapter-specific primers. The RNA-seq libraries were constructed for high-throughput sequencing with the Illumina HiSeq (Illumina Inc., CA, United States) platform to generate paired-end sequencing raw data.

### RNA Sequence Analysis

The quality of raw RNA-seq reads was checked by the FastQC package, and the high-quality clean reads were obtained by independently mapping to the reference genome with the TopHat 2.0 program. The mapped clean reads were counted and normalized into fragments per kilobase of transcript per million fragments mapped reads (FPKM) value using Cufflinks. Differentially expressed genes (DEGs) among the samples were determined using DESeq. For identifying the DAS events, the sequencing data were analyzed by rMATS v4.1.0.^1^ The statistical parameters were defined as false discovery rate (FDR) <0.05 and *p* < 0.05.

### Gene Ontology and Kyoto Encyclopedia of Genes and Genomics Enrichment Analysis

Functional annotations of the DEGs and DAS genes were performed and searched against the NR, Swiss-Prot, Gene Ontology (GO), and Kyoto Encyclopedia of Genes and Genomics (KEGG) databases. Classification and enrichment of DEGs and DAS were carried out by WEGO and ArigGO (Du et al., 2010), respectively. GO and KEGG pathway enrichment analysis was conducted by Fisher’s exact test corrected with a FDR of 5%.

### Quantitative Real-Time-PCR Analysis

Total RNA from 6-month-old seedlings was extracted by the RNAprep Pure Plant Kit (Tiangen Biotech, China). RNase-free DNase I was used to remove potential genomic DNA contaminations. Reverse transcription was determined by M-MLV reverse transcriptase (Invitrogen, MA, United States) with 5 μg quantity of RNA. The synthesized cDNA was used as a template for quantitative real-time-PCR (qRT-PCR) with the primers listed in Supplementary Table 7. The qRT-PCR was carried out with the following protocol: 95°C for 10 min, followed by 40 cycles of 95°C for 15 s, and at 60°C for 30 s. After amplification, the melting curves were used to verify the PCR products. Three independent biological replicates for each sample and three technical replicates for each biological replicate were analyzed. *PdUbiquitin* was used as an internal control. The experimental results were analyzed by the 2^–ΔΔ*CT*^ method (Livak and Schmittgen, 2001).

### Statistical Analyses

All results of the various treatments were based on at least three independent experiments. The values presented are the means ± SD. Statistical analysis was conducted with Student’s *t*-tests for detecting the significance. A value of *p* < 0.05 was considered a statistically significant difference. Double and single asterisks indicate significant differences between comparing samples at 0.01 and 0.05, respectively.

## Results

### Effect of Salinity on the Growth and the Synthesis of Amino Acids

**FIGURE 1:**
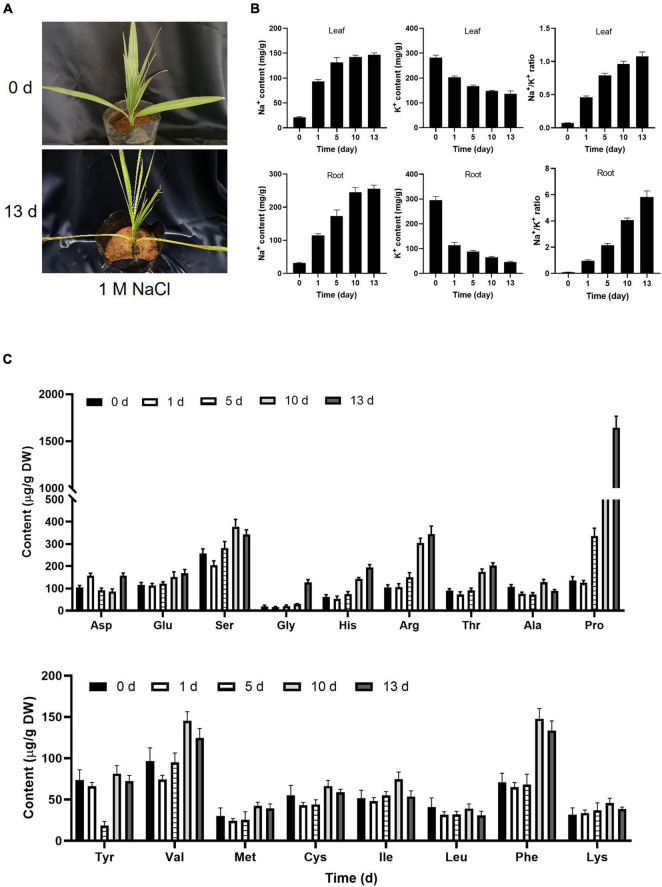
Physiological changes of date palm in response to salt stress. **(A)** Represented images of 6-month-old date palm plants were subjected to salt stress for indicated time. **(B,C)** Concentration of Na^+^ and K^+^ and the content of amino acids in the date palm respond to salt stress. The 6-month-old date palm plants with similar size were subjected to 1 M NaCl solution for indicated time, and the leaves of the date palm trees with the same position were harvested for ion measurement **(B)** and amino acid profiles **(C)**.

A previous study suggested that date palm is a relatively salt-tolerant species that can sustain the growth up to 12.8 dS m^–1^ (Ramoliya and Pandey, 2003). The 6-month-old date palm seedlings were irrigated with different concentrations of NaCl solution every 2 days for 13 days. Consistent with a previous study, no visible changes were observed in the leaf surface with salt-treated seedlings from 200 to 800 mM NaCl for 5 days, while yellowish leaves were observed under 1,000 mM NaCl (Supplementary Figure 1A). Curled leaves were beginning to observe under 500 mM NaCl for 13 days, and apparent yellowish and curled leaves were observed under 1,000 mM NaCl for 13 days (Figure 1A). However, there were no obvious changes in salt-treated seedlings from 200 to 400 mM NaCl for 13 days (Supplementary Figure 1A). Meantime, salt stress could interfere with the homeostasis of ROS which led to ROS accumulation. It was found that the content of H_2_O_2_ was highly accumulated for 5 days, and decreased with prolonged salt stress treatment, and the activities of SOD and POD were also increased in response to salt stress, implying that plants could alleviate the damage of membrane integrity *via* oxidizing membrane lipids that were triggered by ROS (Supplementary Figure 1B). It is well known that plasma membrane was depolarized when plant cells were exposed to a high concentration of salinity stress, and then increased the K^+^ efflux resulted in the K^+^ nutrient deficiency in plants under salt stress (Qiu et al., 2002; Zhang et al., 2018, 2021). Therefore, maintaining the balance between cellular Na^+^ and K^+^ levels in the plant cells was critical for plant growth and salt tolerance under high salinity. Consistent with a previous study (Al-Abdoulhadi et al., 2012), the Na^+^/K^+^ ratio in both root and leaves of date palm trees was increased under high salinity (Figure 1B). These results demonstrated that the high salinity inhibits plant growth by disequilibrating the K^+^/Na^+^ homeostasis in date palm. Moreover, salt stress induced a large increase in the synthesis of amino acids, inhibition of amino acids degradation, inhibition of protein synthesis, and protein degradation that resulted in the accumulation of free amino acids (Zhang et al., 2017; Arif et al., 2020; Che-Othman et al., 2020). Within the amino acids, the content of proline showed the largest increase with 15.2-fold under salt stress treatment for 13 days (Figure 1C). Meantime, the content of arginine, threonine, and histidine showed a significant increase under salt stress treatment for 13 days (Figure 1C). Therefore, the results of amino acids in salt-stressed seedlings suggested that the accumulation of these solutes might have an important role in the tolerance of date palm trees to salt stress.

### Overview of RNA-Seq Data

To elucidate the potential molecular mechanism of date palm in response to salt stress, transcriptome profiles of 6-month-old date palm seedlings treated with or without salt were conducted by mRNA-sequencing (RNA-Seq). As the date palm seedlings were insensitive to salinity (Supplementary Figure 1A), we therefore subjected the seedlings to 1,000 mM NaCl solution for 13 days. A total of 1.7 billion sequenced reads were obtained by using the Illumina RNA-Seq (Supplementary Table 1). Approximately 63.8% of these reads could be uniquely mapped to the date palm reference genome sequence (Supplementary Table 1). About 62.1% of the reads were from exonic regions, whereas 37.9% were mapped to intergenic and intronic regions (Supplementary Figure 2A), which is consistent with the quality of date palm genome assemblies and annotation. Uniform distribution with no obvious 3′/5′bias was observed by plotting the coverage of reads along with each transcript, indicating the high quality of the cDNA libraries (Supplementary Figure 2B). Sequencing saturation assessment showed that more reads were obtained (Supplementary Figure 2C), and 40,786 new transcripts were predicted (Supplementary Table 2), suggesting that extensive coverage was achieved.

### Transcriptome Changes of Date Palm in Response to Salt Stress

**FIGURE 2:**
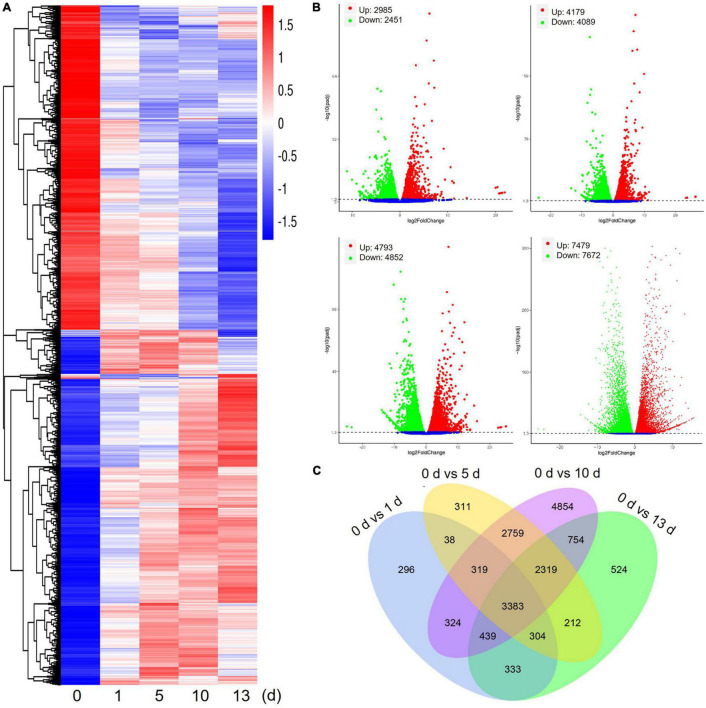
Differentially expressed genes (DEGs) of date palm in response to high salinity from RNA-seq data. **(A)** Heatmap showed expression of increased or decreased trend of total DEGs for indicated time after salt stress treatment. The scaling of each gene to a mean of 0 and SD of 1.5. **(B)** Summary of significant upregulated and downregulated genes caused by high salinity for indicated time in date palm. **(C)** Venn diagram analysis showed a total of 17,169 DEGs in different conditions.

Differentially expressed genes of date palm tree were conducted using DESeq2 software under high salt stress conditions (Figure 2). Transcriptome profiling revealed a large number of DEGs (foldchange ≥ 2 and FDR ≤ 0.05) in salt stress-plants at 1, 5, 10, and 13 days in comparison with control plants (0 day), respectively (Figure 2A). In comparison with control plants, a total of 5,436 DEGs (2,985 upregulated and 2,451 downregulated) were detected after 1 day of salt treatment (0 vs. 1 day), 8,268 DEGs (4,179 upregulated and 4,089 downregulated) were detected after 5 days of salt treatment (0 vs. 5 days), and 9,645 DEGs (4,793 upregulated and 4,852 downregulated) were detected after 10 days of salt treatment (0 vs. 10 days) (Figure 2B). Obviously, the largest number of DEGs (15,151 DEGs, 7,479 upregulated and 7,672 downregulated) were observed after 13 days of salt treatment (0 vs. 13 days). Venn diagram data are expressed using DEGs between salt stress-plants and control plants for the indicated time (Figure 2C). These results suggested that many genes were differentially expressed under high salt stress condition. In addition, the number of shared DEGs among 5, 10, and 13 days were more than that of 1 day (Figure 2C).

**FIGURE 3:**
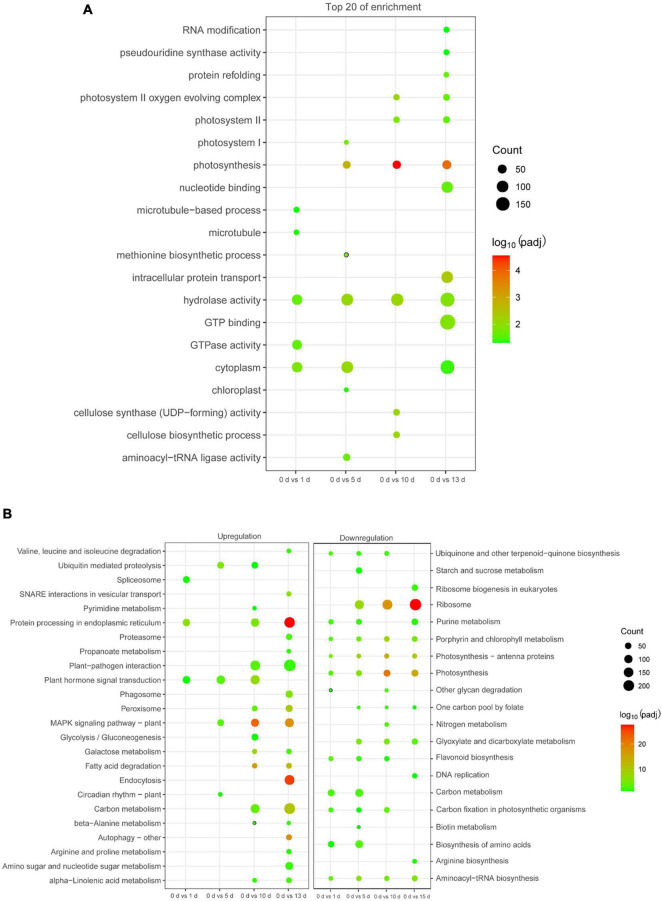
Function enrichment analysis of DEGs in date palm respond to high salinity. **(A)** Gene Ontology (GO) enrichment analysis for the DEGs. **(B)** Kyoto Encyclopedia of Genes and Genomics (KEGG) pathway analysis for the DEGs.

To characterize the functions of the DEGs related to high salinity, GO analysis was conducted in these DEGs. It was clearly shown that diverse functional categories were over-represented in their comparison groups (Supplementary Table 3), and several GO categories, such as “response to metal ion,” “RNA binding,” “potassium ion transmembrane transport,” “calcium ion binding,” and “photosynthesis,” were over-represented (Figure 3A and Supplementary Table 3). We then identified the biological pathway of DEGs associated with high salinity, all DEGs were compared against the KEGG using DAVID with a cut-off probability ≥0.95 and fold change ≥ 2. In total, all DEGs were enriched in 44 different functional pathway categories with 24 categories of upregulation and 20 categories of downregulation in all pairwise comparisons (Figure 3B and Supplementary Table 4). On the category of downregulation, the important biochemical metabolic pathway was “photosynthesis—antenna proteins” and “biosynthesis of amino acids,” while “circadian rhythm—plant” and “MAPK signaling pathway—plant” showed the important biochemical metabolic pathway in the category of upregulation (Supplementary Table 4). Interestingly, the pathway of “spliceosome” was also enriched in the category of upregulation (Supplementary Table 4), indicating their potential role of AS in date palm response to salt stress.

### Identification of Alternative Splicing Events in Response to High Salinity

**FIGURE 4:**
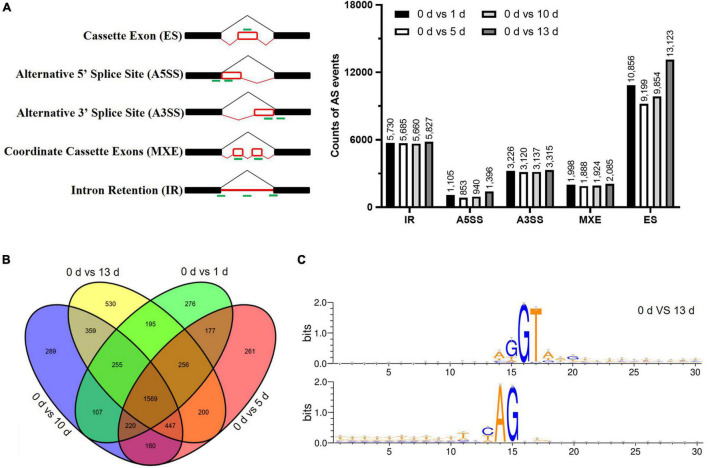
The alternative splicing (AS) profile of date palm was affected by high salinity. **(A)** The differentially alternative splicing (DAS) events caused by high salinity in date palm. **(B)** The Venn diagrams of DAS events caused by high salinity for indicated time in date palm. The overlapped region stands for the DAS events caused by different stress treatment. **(C)** The nucleotide sequences around the alternative 5′SS and 3′SS were shown by Weblogo. The results indicated that these alternative 5′SS and 3′SS were still associated with GU and AG dinucleotides.

Alternative splicing events were identified by using the rMATs software to characterize the five splicing patterns, such as exon skipping (ES), intron retention (IR), alternative 5′ splice site (A5SS), alternative 3′ splice site (A3SS), and mutually exclusive exon (MXE). A total of 90,921 AS events were identified in the four datasets (such as, 43,032 ES, 22,902 IR, 7,895 A5SS, 12,798 A3SS, and 4,294 MXE) under salt stress (Figure 4A and Supplementary Table 5). The numbers of AS events were significantly increased by prolonged salt stress treatment, suggesting that salt prompted the AS of genes which in turn adapt to the environmental stress. There were 1,569 AS genes that were conserved in 5,301 identified AS genes among different stress treatments (Figure 4B). It was found that some genes are AS specific in particular stress treatment, for example, 276, 261, 289, and 530-specific AS genes were found in particular stress treatment (Figure 4B), and the number of stress-specific AS genes at 13 days after salt stress were significantly more than the number of other salt stress treatment, suggesting that salt stress stimulated the novel AS genes in date palm. With regard to the splicing donor-acceptor sites, GU-AG was found to be the canonical among the five different time points (Figure 4C and Supplementary Figure 3), indicating their conserved regulatory roles among plant species.

**FIGURE 5:**
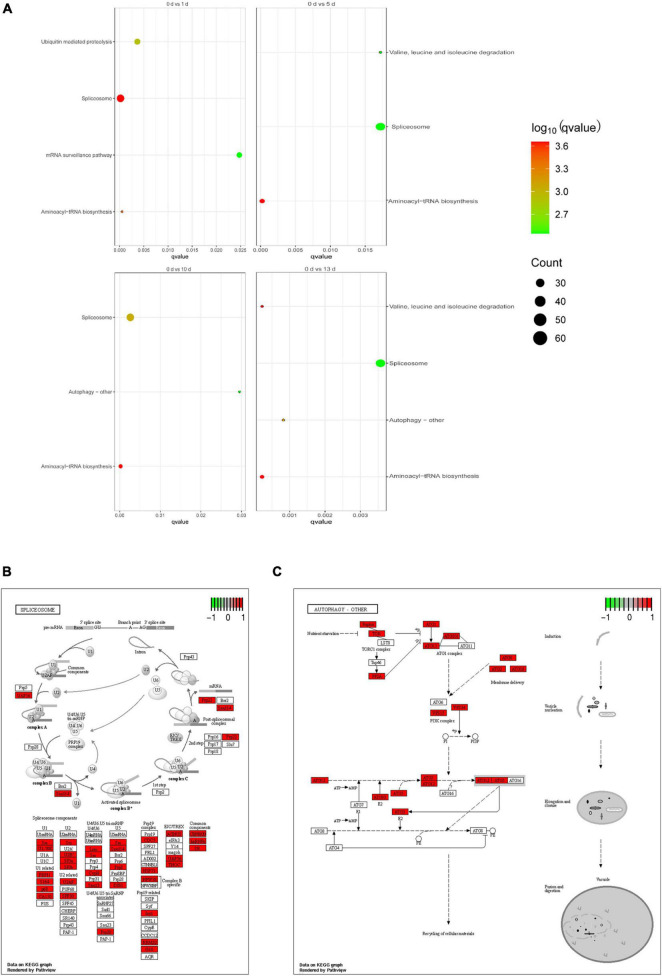
Function enrichment analysis of DAS events of date palm in response to high salinity. **(A)** KEGG pathway analysis for the DAS events of date palm in response to high salinity. **(B)** The pathview of enrichment pathway “Spliceosome” was identified in **(A)**. **(C)** The pathview of enrichment pathway “autophagy-other” was identified in **(A)**.

To further determine the biological functions of AS genes involved in response to salt stress in date palm, we performed GO enrichment analysis of DAS genes in these five time points. The proportions of enriched GO terms for AS genes based on biological process, molecular function, and cellular component were shown according to the *p* (*p* < 0.01) (Supplementary Figure 4). When the date palm plants were initially subjected to salt stress, the DAS genes were mainly enriched on the RNA binding and the metabolic process of RNA and tRNA (Supplementary Figure 4). Interestingly, pathways relating to amino acid, photosystem II, and ligase activity were significantly enriched under salt stress conditions. Moreover, the DAS genes were annotated for KEGG pathways (Supplementary Figure 5). The enrichment pathway analysis showed that these genes were significantly enriched in six pathways, such as spliceosome, aminoacyl-tRNA biosynthesis, mRNA surveillance pathway, ubiquitin-mediated proteolysis, valine, leucine and isoleucine degradation, and autophagy—other (Figure 5A). In the spliceosome pathway, several proteins related to the U1, U2, U4/U6, U5 snRNP, and splicing factors were observed (Figure 5B), indicating that those proteins were participated in the regulation of AS of target genes upon salt stress in the date palm. Interestingly, several autophagy-related proteins have been differentially alternative spliced in response to salt stress (Figure 5C), suggesting that the autophagy pathway might be participated in the regulation of salt tolerance in date palm.

### Overlapping and Distinct Role of Differentially Alternative Splicing and Differentially Expressed Gene in Response to High Salinity

**FIGURE 6:**
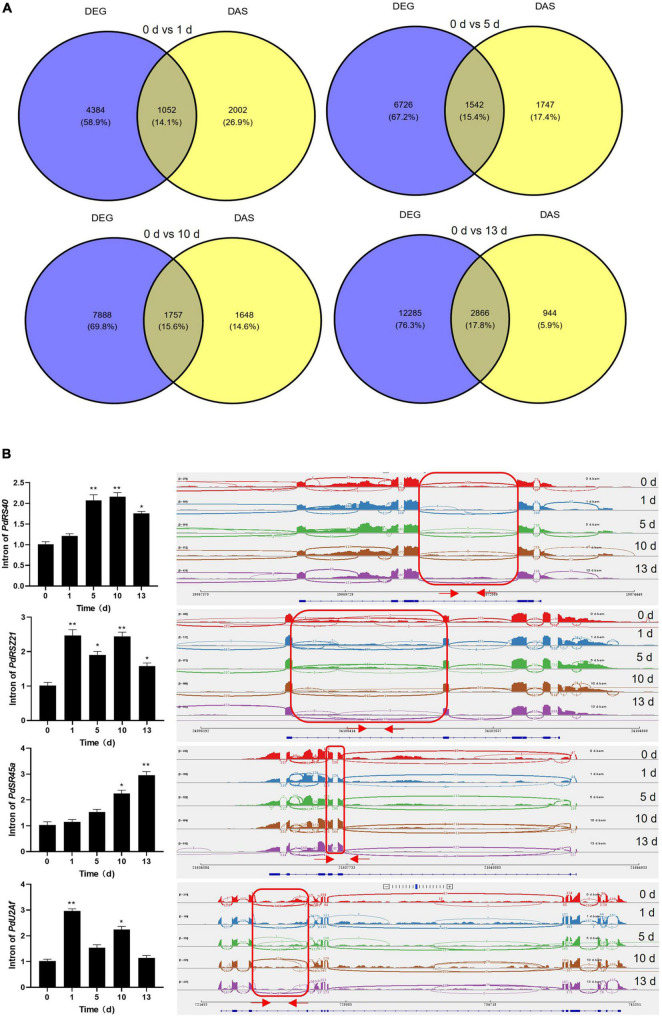
Categorization of DEGs and DAS events and validation of DAS events. **(A)** Venn diagram of the overlap of DEGs and DAS events. **(B)** Validation of DAS events which were related to splicing factors by quantitative real-time-PCR (qRT-PCR). qRT-PCR analysis was performed for four splicing factors in date palm for indicated time under salt stress condition (1 M NaCl). The expression of *PdUbiquitin* was used as the internal control. Red arrows in the right panel showed the primers position of each intron. Error bar means ± SD (*n* = 3). Asterisks indicate significant differences (*p < 0.05 and **p < 0.01).

Previous studies showed that the genes with the function of the salt stress response are modulated transcriptionally and post-transcriptionally in plant (Feng et al., 2015; Shang et al., 2017; Ganie and Reddy, 2021; Martin et al., 2021; Zhang et al., 2021). It was observed that a subset of genes was overlapped between DAS and DEGs datasets among four stress treatments (Figure 6A). Moreover, the number of overlapped genes was increased with prolonged salt stress treatment, and GO analysis revealed that these genes were enriched in several important functional pathways, such as “RNA binding,” “amino acid activation,” “tRNA metabolic process,” and “photosystem II” (Supplementary Figure 6). Meantime, KEGG pathways showed that several critical pathways were significantly enriched, such as aminoacyl-tRNA biosynthesis, glycerolipid metabolism, glyoxylate and dicarboxylate metabolism, porphyrin and chlorophyll metabolism, valine, leucine and isoleucine degradation, and autophagy–other, indicating their separate regulation of gene expression and AS in response to salt stress (Supplementary Figure 7). These results suggested that several genes with salt-related function were manipulated by both transcriptionally and post-transcriptionally in date palm. Moreover, the number of DAS-specific genes was gradually decreased while the number of DEG-specific genes was increased with prolonged salt stress treatment (Figure 6A), suggesting that AS and gene expression could be distinctively regulated by salt stress. Actually, KEGG analysis confirmed that the over-represented functional categories largely differed between the DAS and DEGs, such as “RNA binding” and “RNA processing,” were only over-represented among DAS genes (Supplementary Figure 8), while “photosynthesis,” “amino acid biosynthesis,” and “transporter activity” were found among the DEGs group (Supplementary Figure 9), indicating their independent regulation of gene expression and AS in response to salt stress.

### Validation of Alternative Splicing Events and Gene Expression by Quantitative Real-Time-PCR

To validate the DAS events and DEGs that were predicted by bioinformatic analysis, qRT-PCR was carried out on the genes that produced different types of AS events and different gene expressions upon salt stress treatment. It was shown that the DAS genes were mainly enriched on the RNA binding in date palm response to salt stress (Supplementary Figure 4), and evidence indicated that splicing factors modulate stress responses which unveil a novel regulatory layer in plant stress adaptation (Laloum et al., 2018). We therefore chose four splicing factors that were aberrantly spliced under salt stress conditions in date palm. The qRT-PCR analysis showed that the splicing efficiencies of these splicing factors were decreased when date palm plants were subjected to salt stress (Figure 6B and Supplementary Table 5), and the IR intensity of *PdSR45a* gene was increased with prolonged salt stress treatment. While other three genes, *PdRS40*, *PdRSZ21*, and *PdU2Af*, the IR intensity was increased up to 10 days, and decreased at 13 days after salt stress treatment (Figure 6B), suggesting that AS of these splicing factors might affect the isoforms of downstream genes that contributed to salt stress tolerance in date palm. Moreover, transcript levels of *PdSOS2;1*, *PdSOS2;2*, *PdSOS4*, *PdSOS5*, and *PdCIPK11* were significantly induced while the expression of *PdFAD4* was decreased in response to salt stress, which is consistent with RNA-seq data (Supplementary Figure 10 and Supplementary Table 6), indicating that these genes are involved in the regulation of salt stress response in date palm, and the bioinformatic prediction based on the primary sequencing data was reliable.

## Discussion

Date palm grows in arid and semi-arid areas where soil contains a high concentration of salts, supposed to be a salt-tolerant plant, and have evolved a series of mechanism for their adaptation. However, the mechanism for their adaptation to salt stress was largely unknown. Several studies have been performed in date palm for the identification of mRNAs, metabolites, and proteomic in response to salt stress (Yaish et al., 2017; Jana et al., 2019; Al Kharusi et al., 2021; Sghaier-Hammami et al., 2021). As an important molecular regulation layer, the involvement of post-transcriptional mechanism was still poorly understood under salt stress conditions in date palm. In the present study, we investigated the genome-wide of transcriptional changes and AS events under salt stress conditions in date palm plant through high-throughput RNA-seq. Amounts of DEGs were identified under salt stress in comparison with previous studies (Radwan et al., 2015; Yaish et al., 2017). Importantly, many DAS events were found in response to salt stress, and some splicing factors were abnormally spliced under salinity, suggesting that AS-related proteins might be involved in regulating the salt stress pathway. Therefore, our study highlighted the pivotal role of AS in the regulation of salt stress and provided novel insights for enhancing the resistance to salt in date palm.

Plant has evolved several mechanisms to deal with high salinity, such as regulating the ion influx and efflux at the plasma membrane, ion compartmentation in vacuoles, and maintaining the osmotic balance, and coping with the excessive amounts of salt in cells by generating additional quantities of antioxidants (Zhu, 2016; Yang and Guo, 2018b; Zhang et al., 2021). Previous studies showed that the homeostasis between cellular Na^+^ and K^+^ levels played pivotal role on the plant growth and development in the saline environments (Yang and Guo, 2018a; Zhang et al., 2018; Ma et al., 2019; Yang et al., 2019). Consistent with these previous studies, salt treatment increased the ratio of Na^+^/K^+^ in the date palm plants (Figure 1B). It was known that the SOS pathway plays important role in sustaining the Na^+^/K^+^ homeostasis resulted in resistance to salt stress in plants (Zhu, 2016; Zhang et al., 2021). The *Arabidopsis* SOS3 interacted with and activated the SOS2 to form the SOS2–SOS3 complex (Halfter et al., 2000), and the complex phosphorylated and activated the SOS1 to remove Na^+^ from the cytosol (Qiu et al., 2002). Moreover, environmental signals generate the Ca^2+^ signals, and then sensed by Ca^2+^ sensors, such as calmodulin (Saimi and Kung, 2002), CaM-related proteins, and calcium-depended protein kinases (CDPKs) (Yang and Poovaiah, 2003). Upon stress treatment, the expression levels of *PdSOS2;1*, *PdSOS2;2*, *PdSOS4*, *PdSOS5*, and *PdCIPK11* were significantly induced in date palm when compared with the plants under normal conditions (Supplementary Figure 5), indicating that high salinity affected the Na^+^/K^+^ homeostasis resulted in the inhibition of plant growth which might depend on the SOS pathway. Additionally, metabolome analysis revealed that various amino acids were reported to change in response to salt stress (Al Kharusi et al., 2021), and the content of some amino acids was increased with prolonged salt stress treatment (Figure 1C), suggesting that salt stress caused a large increase in the synthesis of amino acids to alleviate the stress damage to date palm plant. Metabolomic analysis of date palm seedlings exposed to salinity further showed that the differentially accumulated amino acids might determine the plant growth and salinity tolerance in date palms (Jana et al., 2019).

Alternative splicing regulates the gene expression and broadens the proteome diversity in eukaryotic (Graveley, 2001). AS modulates the gene expression during plant development and in response to abiotic stresses (Graveley, 2001; Syed et al., 2012; Martin et al., 2021). It was clearly shown that the number of AS of genes was increased by salt in date palm (Figure 4), which is consistent with previous studies in several species (Palusa et al., 2007; Ding et al., 2014). AS is regulated by the transacting factors, such as the serine/arginine-rich family of nuclear phosphoproteins (SR proteins), snRNPs, and heterogeneous nuclear ribonucleoproteins (hnRNPs) (Syed et al., 2012; Shang et al., 2017; Martin et al., 2021; Zhang et al., 2021). Manipulation of SR proteins might affect the splicing efficiencies of target genes and other splicing factors (Albaqami et al., 2019; Li et al., 2021; Weng et al., 2021). It was known that the modulation of SR gene isoforms by salt might destabilize the spliceosome complex to accurately recognize the splice sites (Albaqami et al., 2019; Laloum et al., 2021). Our previous studies showed that salt stress promotes the alternative 5′ and 3′ splice-site selection and IR events which led to introduce premature termination (nonsense) codons (PTCs) in the pre-mRNA of SR proteins (Ding et al., 2014; Gu et al., 2020; Hu et al., 2021). Consistent with these studies, several SR proteins were abnormally spliced under high salinity in date palm (Figure 6B). Several studies have been reported in the plant SR proteins (Duque, 2011; Richardson et al., 2011). Recent findings revealed that *AtSR45a* was involved in regulating the plant salinity tolerance at the post-transcriptional level (Albaqami et al., 2019). In addition, AtSR45a interacted with U1-70K and U2AF35b to accurately recognize the splice sites (Golovkin and Reddy, 1999), and *AtSR45a* regulated the AS of 5PTase13 pre-mRNA and the degradation of SnRK1 in response to sugars (Carvalho et al., 2016). Moreover, AtSR45a interacted with SNW/Ski-interacting protein (SKIP) to accurately recognize or cleavage the splice sites, and then the *skip* mutant exhibited the hypersensitive phenotype in response to salt and osmotic stress (Wang et al., 2012). FIERY2/CTD phosphatase-like 1 (FRY2/CPL1) interacted with AtRS40 and AtRS41 proteins, and the loss-of-function mutations showed the hypersensitive phenotype in response to salt and ABA (Chen et al., 2013). Ectopic overexpression of cassava SCL30A and SR34 proteins could improve the salt tolerance in plants (Gu et al., 2020; Hu et al., 2021). Therefore, extensive analysis of SR proteins would provide novel insights on their essential role for proper gene expression underlying salt stress in date palms.

Collectively, we have performed a genome-wide survey of salinity-induced changes in AS events and transcriptome abundance in date palm. A large number of DEGs and DAS were identified using a next-generation sequencing technology. Many of these regulatory genes were belonged to different signaling pathway and metabolism process, indicating their critical roles in response to salt stress. Furthermore, overlapping and distinct roles of DAS and DEGs might be participated in the regulation of salt tolerance to date palm. Importantly, several splicing factors were abnormally spliced under salt, suggesting that these splicing factors might be involved in regulating the RNA splicing and salt tolerance in date palm. Therefore, our finding improved our understanding of transcriptional and post-transcriptional regulatory on improving salt tolerance in date palm and opened the door for helping plant breeders to develop salt-tolerant plants.

## Data Availability Statement

The datasets presented in this study can be found in online repositories. The names of the repository/repositories and accession number(s) can be found below: NGDC GSA Bioproject, accession no: PRJCA007469.

## Author Contributions

Z-YW conceived and designed the experiments and wrote and revised the manuscript. NZ and ZX performed the experiments. NZ, ZX, QW, QL, JW, WA, YL, CL, and MW analyzed and interpreted the data and results. All authors have read and approved the manuscript.

## Conflict of Interest

The authors declare that the research was conducted in the absence of any commercial or financial relationships that could be construed as a potential conflict of interest.

## Publisher’s Note

All claims expressed in this article are solely those of the authors and do not necessarily represent those of their affiliated organizations, or those of the publisher, the editors and the reviewers. Any product that may be evaluated in this article, or claim that may be made by its manufacturer, is not guaranteed or endorsed by the publisher.

## Footnotes

1
http://rnaseq-mats.sourceforge.net
