# Supplementary material for: Salt Stress Modulates the Landscape of Transcriptome and Alternative Splicing in Date Palm (Phoenix dactylifera L.)
Source: Front Plant Sci. 2022 Jan 20;12:807739. doi: 10.3389/fpls.2021.807739 (PMC8810534; doi:10.3389/fpls.2021.807739)
Supplement: Supplementary file 8 [file Presentation_1.PPTX]

## Slide 1
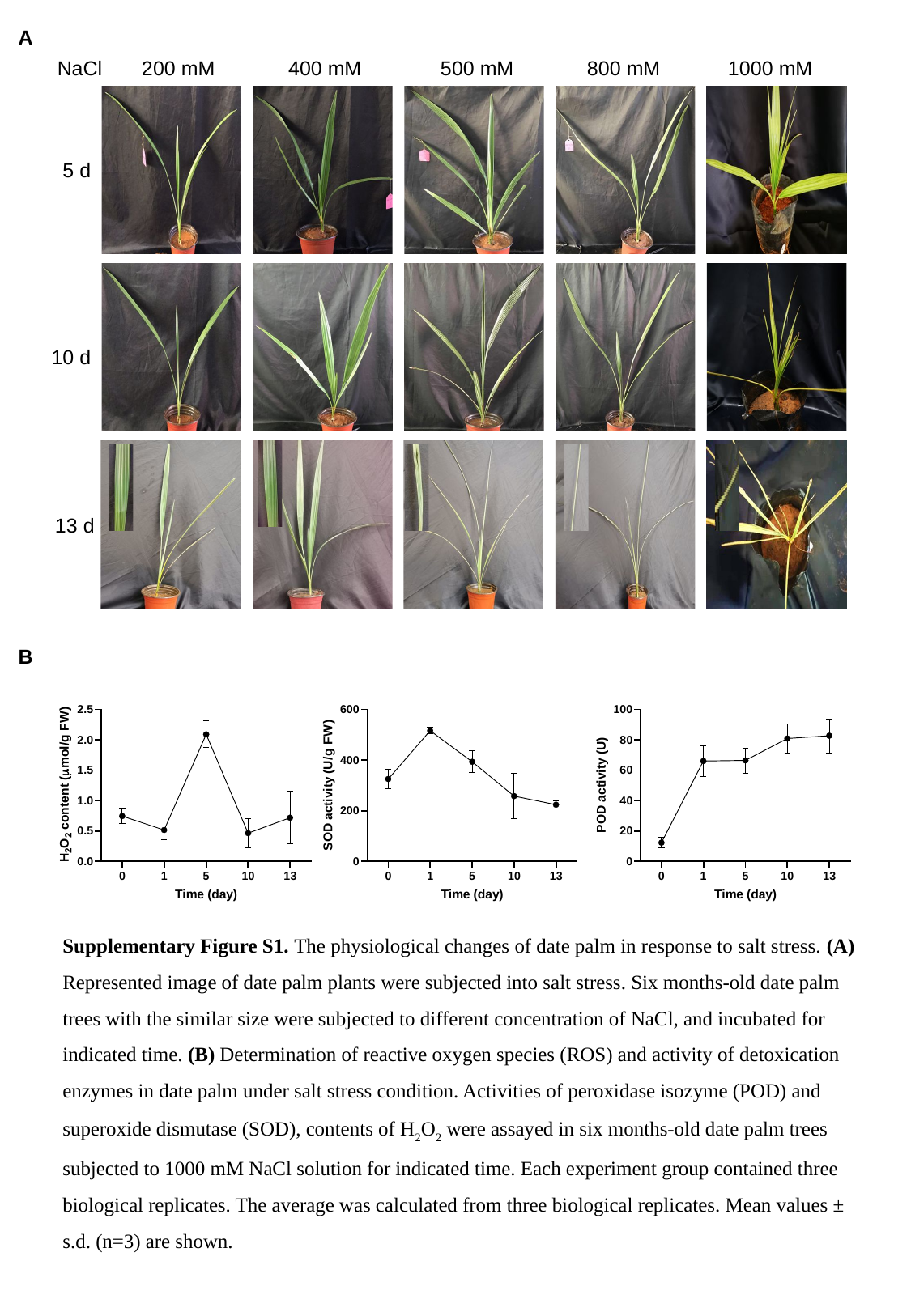

A
NaCl 200 mM 400 mM 500 mM 800 mM 1000 mM
5 d
10 d
13 d
B
Supplementary Figure S1. The physiological changes of date palm in response to salt stress. (A) Represented image of date palm plants were subjected into salt stress. Six months-old date palm trees with the similar size were subjected to different concentration of NaCl, and incubated for indicated time. (B) Determination of reactive oxygen species (ROS) and activity of detoxication enzymes in date palm under salt stress condition. Activities of peroxidase isozyme (POD) and superoxide dismutase (SOD), contents of H2O2 were assayed in six months-old date palm trees subjected to 1000 mM NaCl solution for indicated time. Each experiment group contained three biological replicates. The average was calculated from three biological replicates. Mean values ± s.d. (n=3) are shown.

## Slide 2
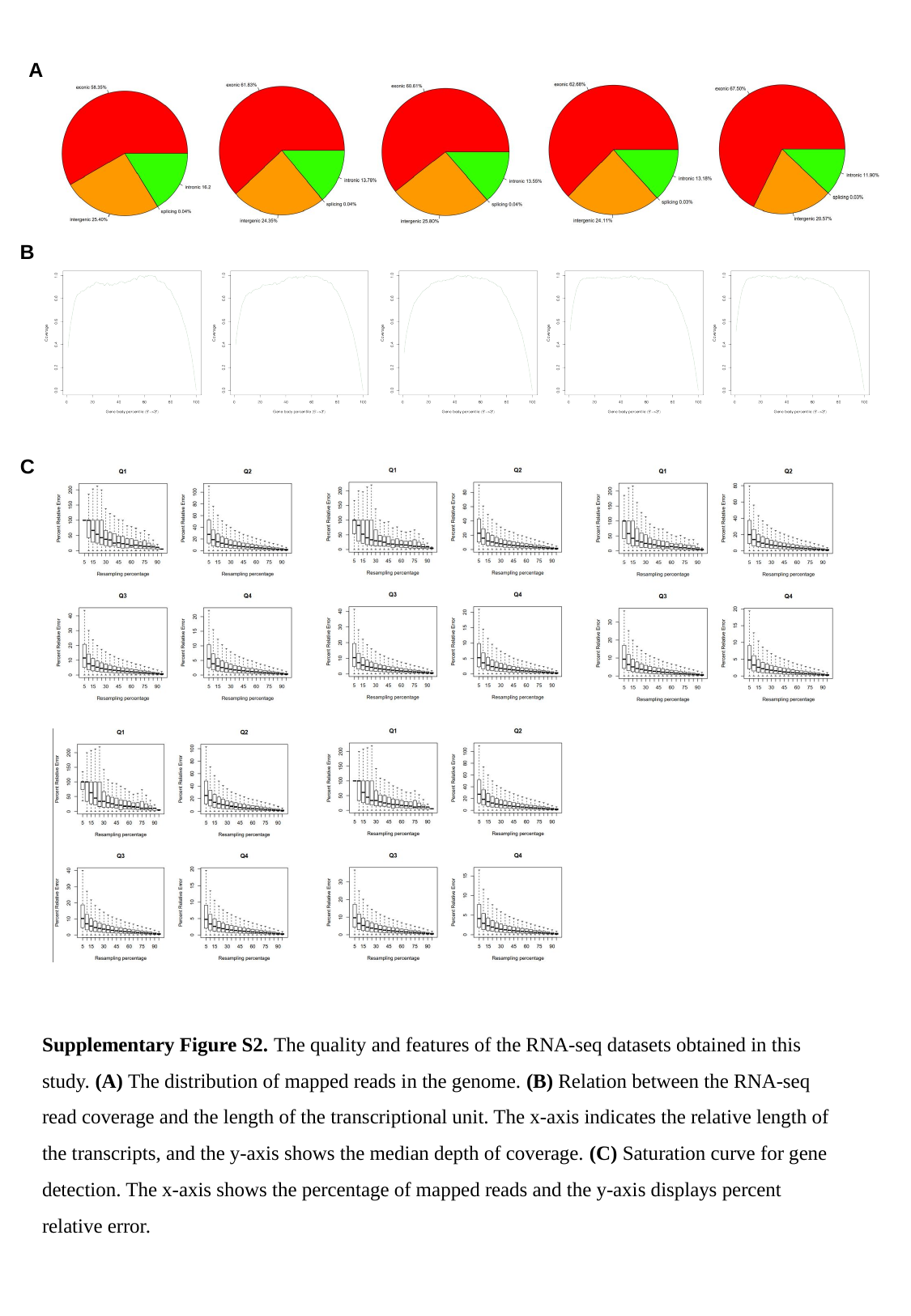

A
B
C
Supplementary Figure S2. The quality and features of the RNA-seq datasets obtained in this study. (A) The distribution of mapped reads in the genome. (B) Relation between the RNA-seq read coverage and the length of the transcriptional unit. The x-axis indicates the relative length of the transcripts, and the y-axis shows the median depth of coverage. (C) Saturation curve for gene detection. The x-axis shows the percentage of mapped reads and the y-axis displays percent relative error.

## Slide 3
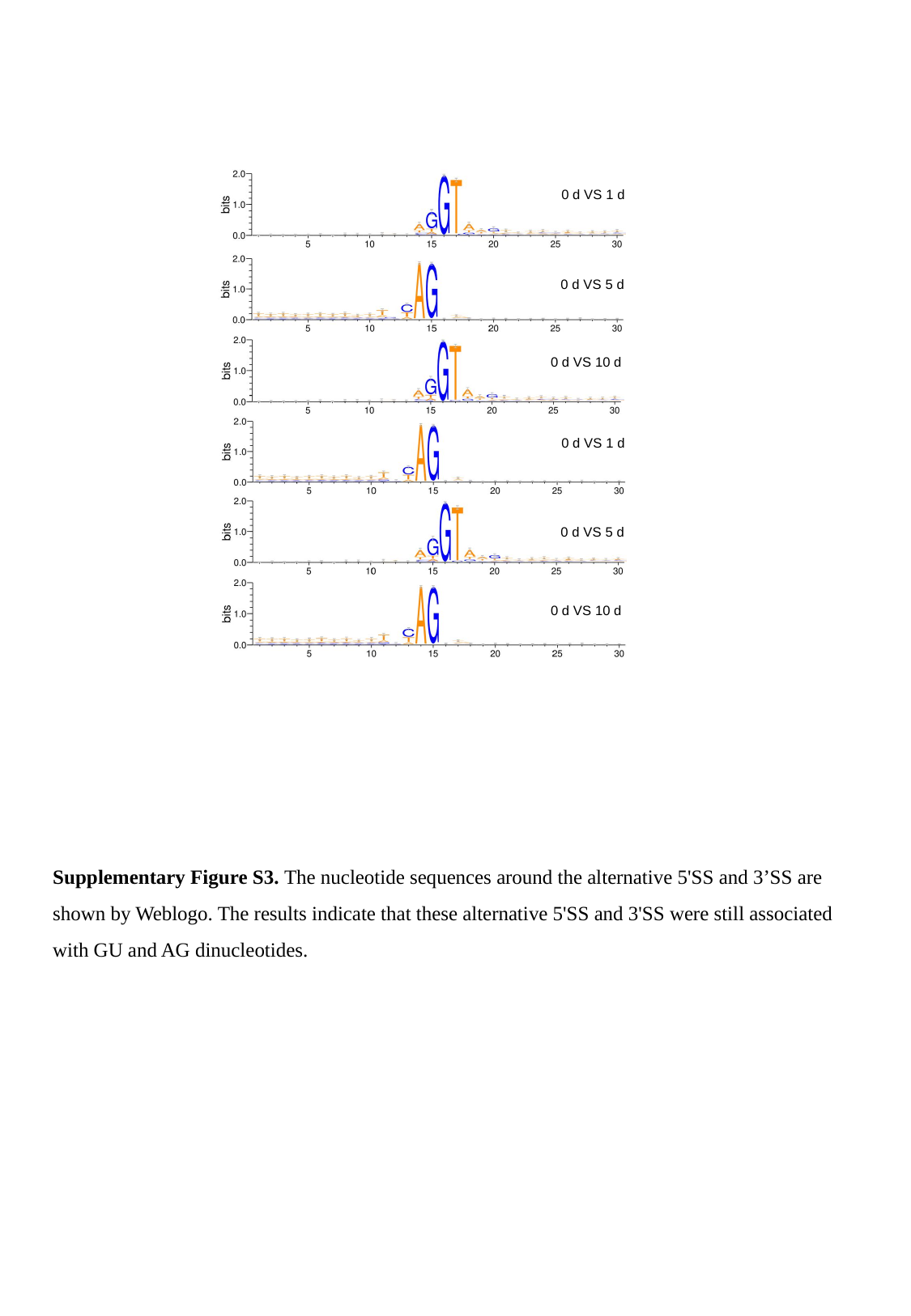

0 d VS 1 d
0 d VS 5 d
0 d VS 10 d
0 d VS 1 d
0 d VS 5 d
0 d VS 10 d
Supplementary Figure S3. The nucleotide sequences around the alternative 5'SS and 3’SS are shown by Weblogo. The results indicate that these alternative 5'SS and 3'SS were still associated with GU and AG dinucleotides.

## Slide 4
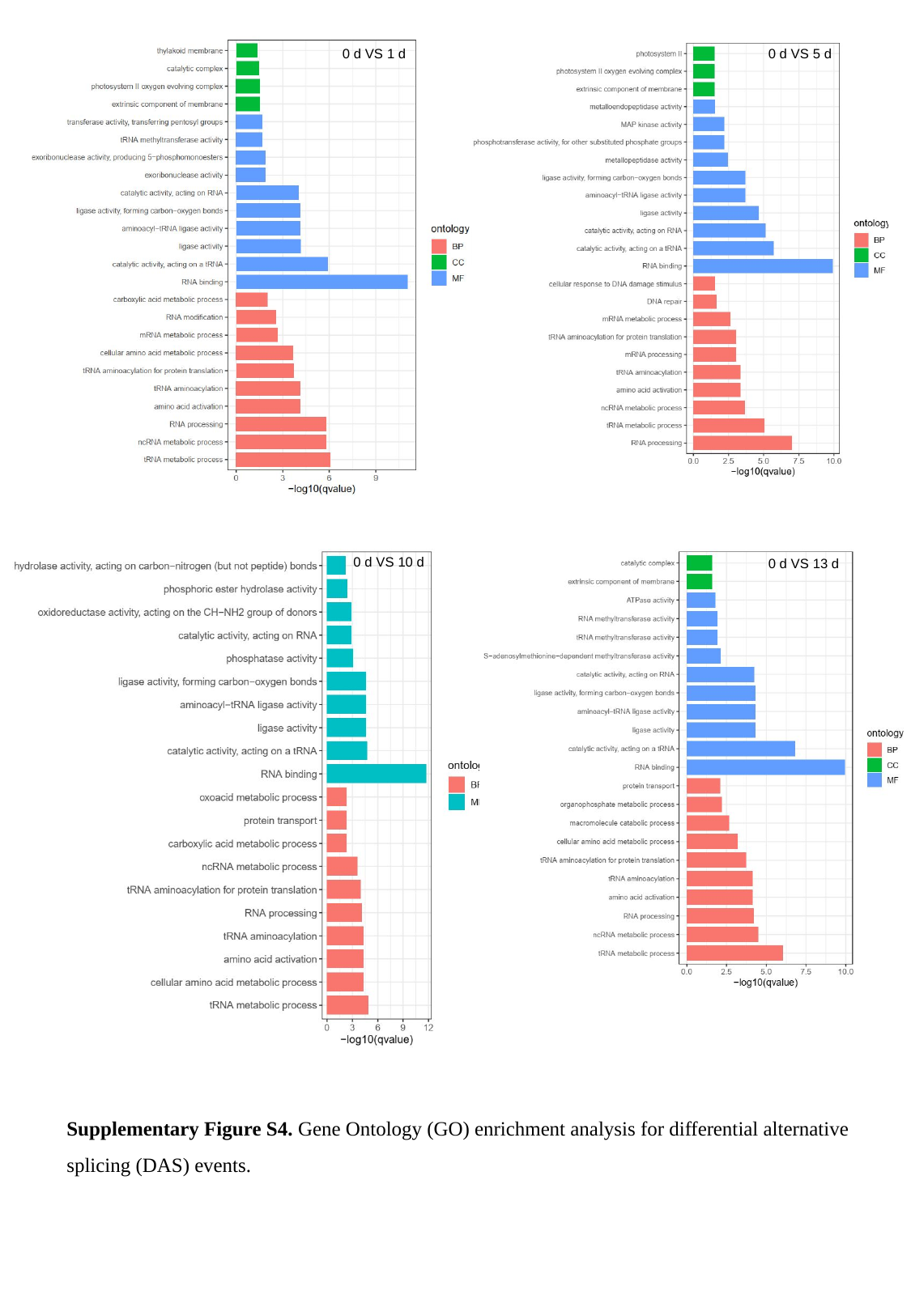

0 d VS 1 d
0 d VS 5 d
0 d VS 10 d
0 d VS 13 d
Supplementary Figure S4. Gene Ontology (GO) enrichment analysis for differential alternative splicing (DAS) events.

## Slide 5
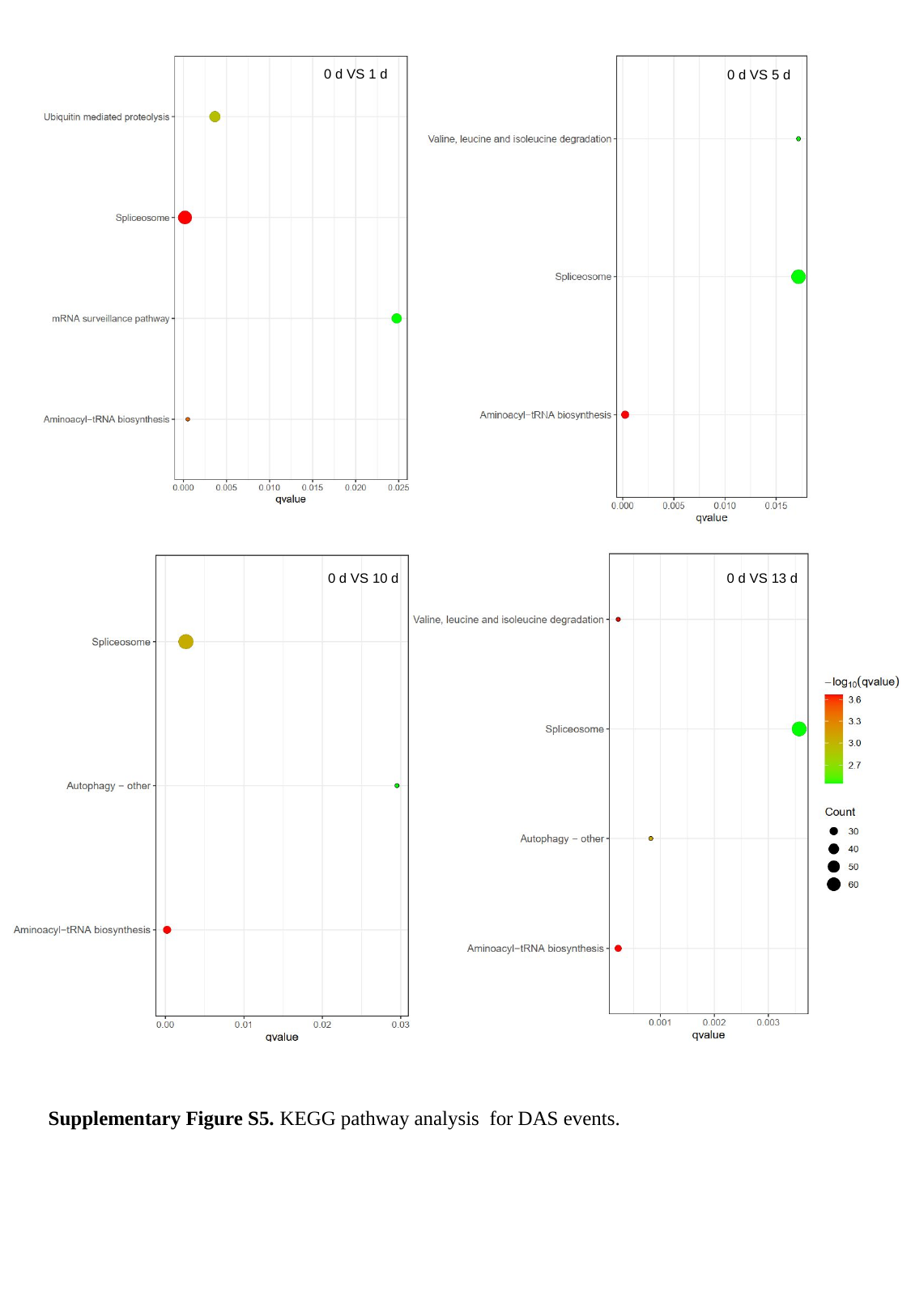

0 d VS 1 d
0 d VS 5 d
0 d VS 10 d
0 d VS 13 d
Supplementary Figure S5. KEGG pathway analysis for DAS events.

## Slide 6
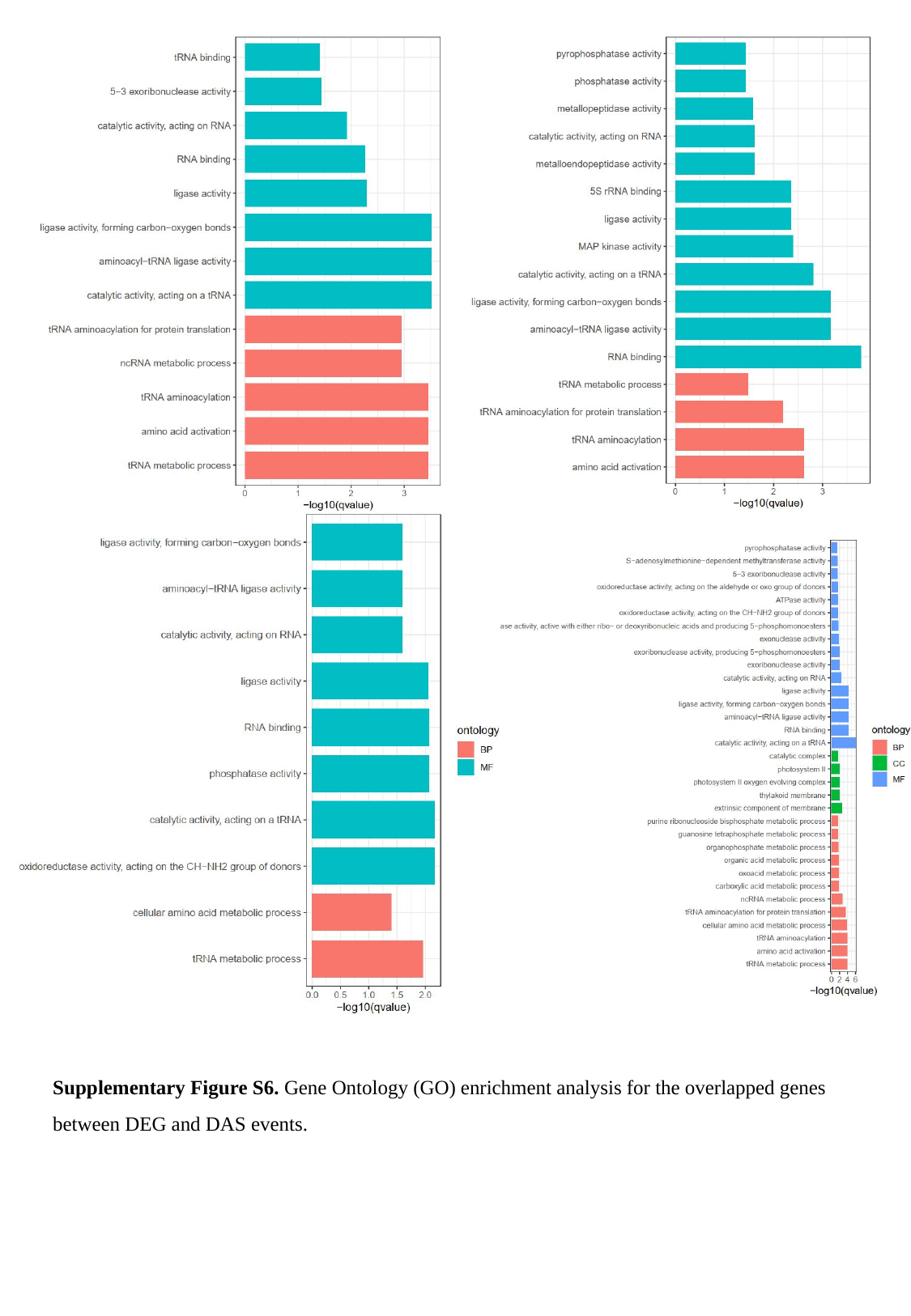

Supplementary Figure S6. Gene Ontology (GO) enrichment analysis for the overlapped genes between DEG and DAS events.

## Slide 7
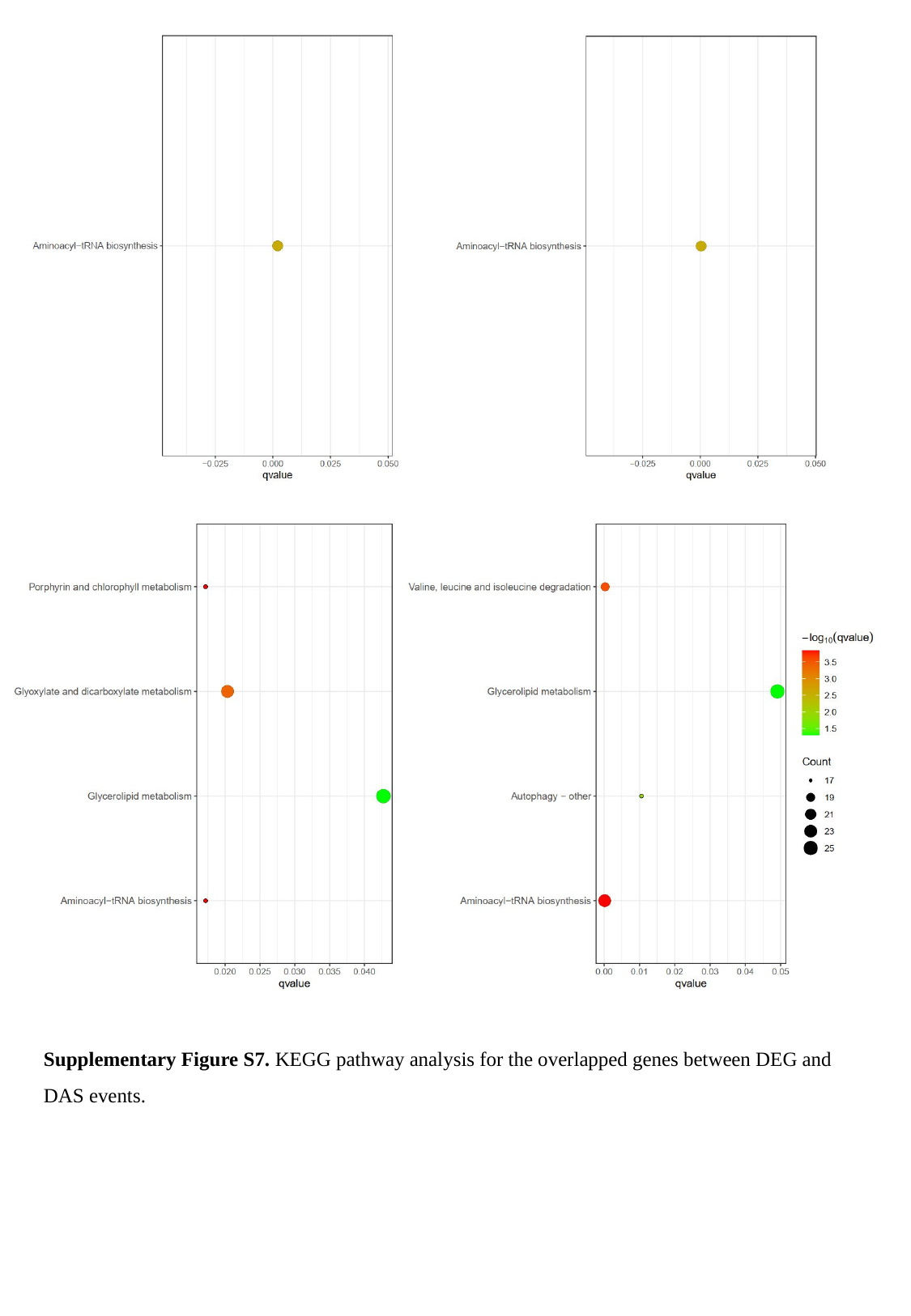

Supplementary Figure S7. KEGG pathway analysis for the overlapped genes between DEG and DAS events.

## Slide 8
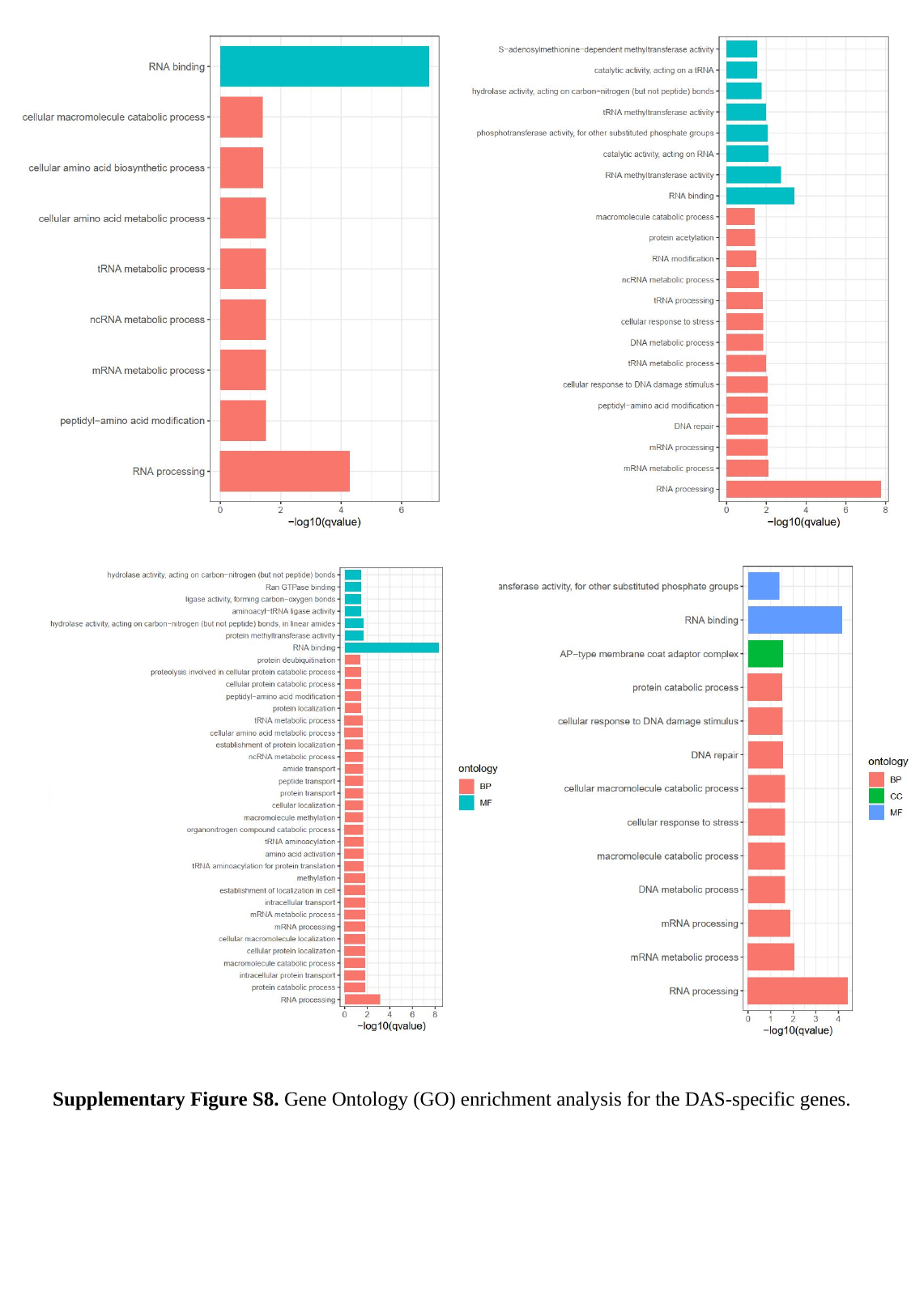

Supplementary Figure S8. Gene Ontology (GO) enrichment analysis for the DAS-specific genes.

## Slide 9
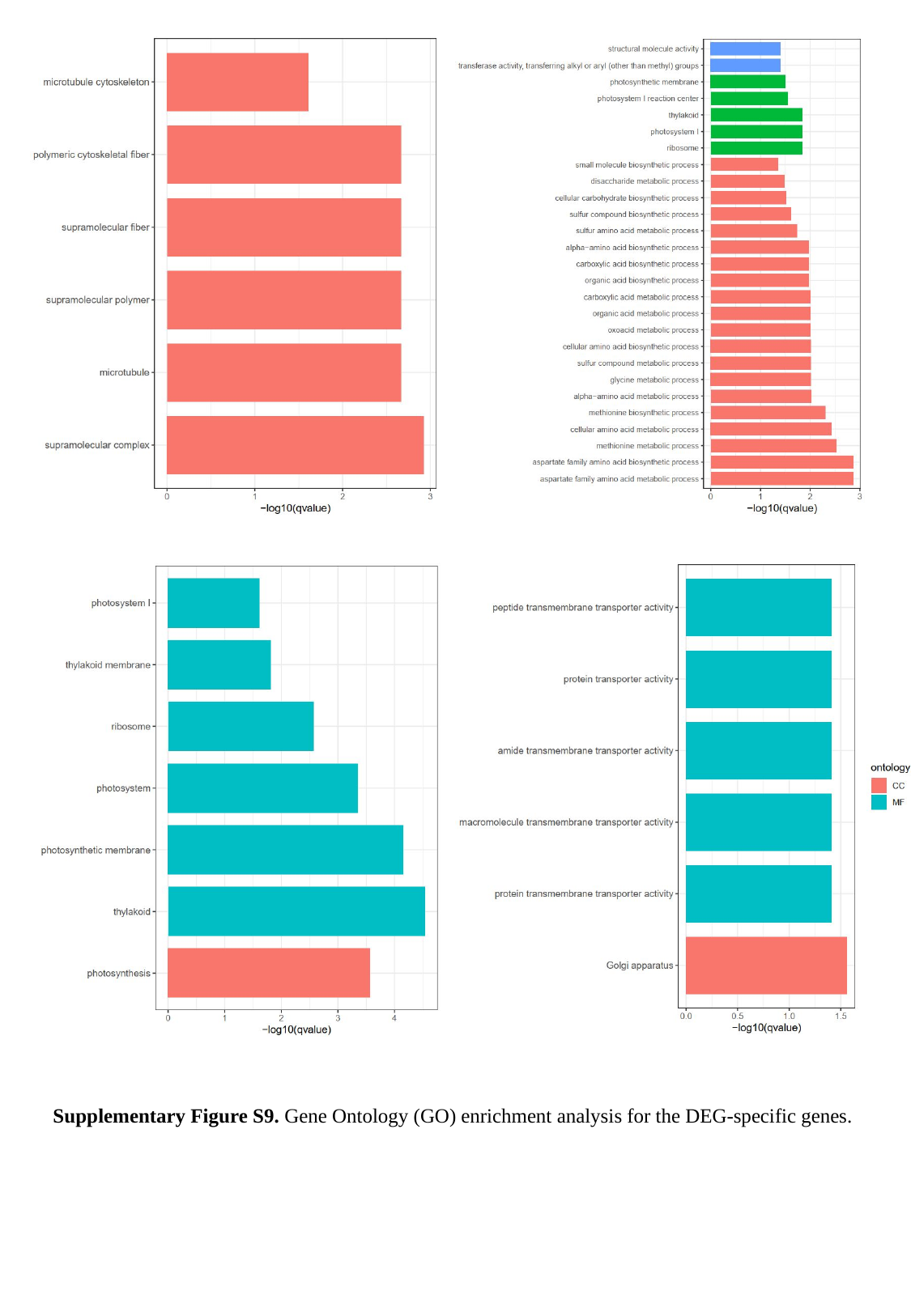

Supplementary Figure S9. Gene Ontology (GO) enrichment analysis for the DEG-specific genes.

## Slide 10
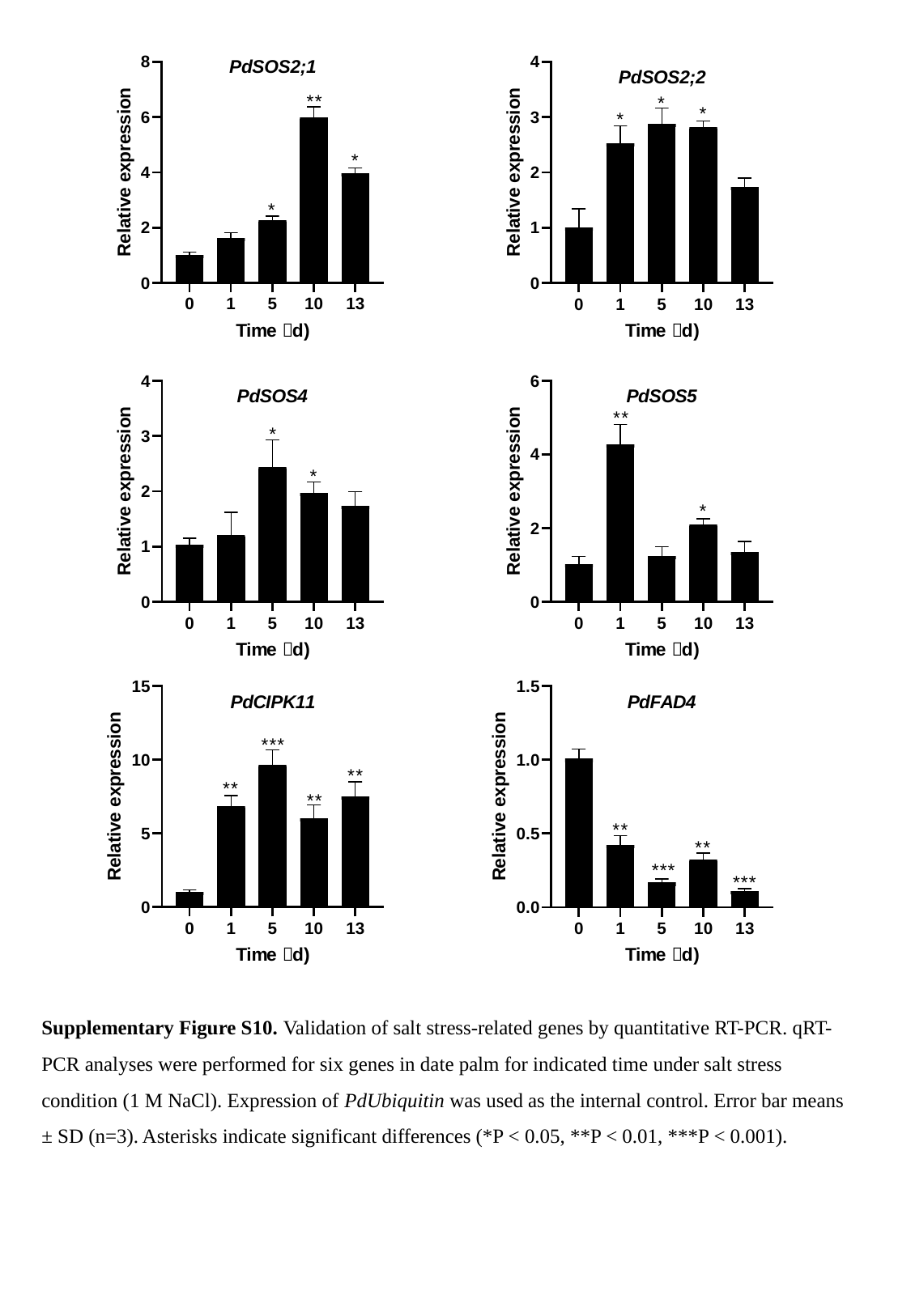

Supplementary Figure S10. Validation of salt stress-related genes by quantitative RT-PCR. qRT-PCR analyses were performed for six genes in date palm for indicated time under salt stress condition (1 M NaCl). Expression of PdUbiquitin was used as the internal control. Error bar means ± SD (n=3). Asterisks indicate significant differences (*P < 0.05, **P < 0.01, ***P < 0.001).
